# PeptiHub: a curated repository of precisely annotated cancer-related peptides with advanced utilities for peptide exploration and discovery

**DOI:** 10.1093/database/baae092

**Published:** 2024-09-20

**Authors:** Sara Zareei, Babak Khorsand, Alireza Dantism, Neda Zareei, Fereshteh Asgharzadeh, Shadi Shams Zahraee, Samane Mashreghi Kashan, Shirin Hekmatirad, Shila Amini, Fatemeh Ghasemi, Maryam Moradnia, Atena Vaghf, Anahid Hemmatpour, Hamdam Hourfar, Soudabeh Niknia, Ali Johari, Fatemeh Salimi, Neda Fariborzi, Zohreh Shojaei, Elaheh Asiaei, Hossein Shabani

**Affiliations:** Department of Cell & Molecular Biology, Faculty of Biological Sciences, Kharazmi University, South Mofateh Ave. , Tehran 15719-14911, Iran; Department of Neurology, University of California, 200 S. Manchester Ave., Suite 206 Orange, Irvine, CA 92868-4280, USA; Department of Computer Engineering, Faculty of Engineering, Ferdowsi University of Mashhad, Azadi Square , Mashhad 9177948974, Iran; Department of Biophysics, Faculty of Biological Sciences, Tarbiat Modares University, Jalal AlAhmad HWY, Tehran 14115-111, Iran; Transplant Research Center, Shiraz University of Medical Sciences, Khalili Str, Shiraz 7193711351, Iran; Department of Medical Physiology, Faculty of Medicine, Mashhad University of Medical Sciences, Azadi Sq., Mashhad 9177948564, Iran; Faculty of Life Sciences and Biotechnology, Shahid Beheshti University, Dr. Shahriari Sq., Tehran 1983969411, Iran; Department of Medicinal Biotechnology, Faculty of Advanced Technology in Medicine, Golestan University of Medical Sciences, Shast Kola Road, Gorgan 4918936316, Iran; Department of Toxicology and Pharmacology, Faculty of Pharmacy, Tehran University of Medical Sciences, 16 Azar Ave, Tehran 1416753955, Iran; Department of Genetics, Faculty of Advanced Science and Technology, Medical Sciences Branch, Islamic Azad University, Shariati St., Tehran 19395/1495, Iran; Department of Computer Engineering, Faculty of Engineering, Ferdowsi University of Mashhad, Azadi Square , Mashhad 9177948974, Iran; Division of Occupational and Environmental Medicine, Department of Laboratory Medicine, Faculty of Medicine, Lund University, Lund BOX 117,221 00, Sweden; Department of Medical Biotechnology, Faculty of Advanced Technologies, Shahrekord University of Medical Science, Kashani BLVD., Shahrekord 8815713471, Iran; Department of Clinical Biochemistry, Faculty of Medicine, Shahid Sadoughi University of Medical Sciences and Health Services, Aalam Sq., Yazd 8915173149, Iran; Bioprocess Engineering Research Group, Department of Industrial and Environmental Biotechnology, National Institute for Genetic Engineering and Biotechnology, Tehran-Karaj HWY, Tehran 14965/161, Iran; Department of Biology, Kavian Institute of Higher Education, Elahiyeh Blv., Mashhad 91863-74915, Iran; Department of Biology, Kavian Institute of Higher Education, Elahiyeh Blv., Mashhad 91863-74915, Iran; Department of Clinical Science, Faculty of Veterinary Medicine, Razi University, Taq-e Bostan, Kermanshah 6714414971, Iran; Department of Biology and Biotechnology, Faculty of Molecular Biology and Genetics, University of Pavia, S.da Nuova, Pavia 65, 27100, Italy; Department of Cell & Molecular Biology, Faculty of Biological Sciences, Kharazmi University, South Mofateh Ave. , Tehran 15719-14911, Iran; Systems Biotechnology Research Group, Department of Industrial and Environmental Biotechnology, National Institute for Genetic Engineering and Biotechnology, Tehran-Karaj HWY., Tehran 14965/161, Iran; Department of Biology, Faculty of Biosciences, Tehran North Branch, Islamic Azad University, Vafadar Blv., Tehran 1651153311, Iran

## Abstract

Peptihub (https://bioinformaticscollege.ir/peptihub/) is a meticulously curated repository of cancer-related peptides (CRPs) that have been documented in scientific literature. A diverse collection of CRPs is included in the PeptiHub, showcasing a spectrum of effects and activities. While some peptides demonstrated significant anticancer efficacy, others exhibited no discernible impact, and some even possessed alternative non-drug functionalities, including drug carrier or carcinogenic attributes. Presently, Peptihub houses 874 CRPs, subjected to evaluation across 10 distinct organism categories, 26 organs, and 438 cell lines. Each entry in the database is accompanied by easily accessible 3D conformations, obtained either experimentally or through predictive methodology. Users are provided with three search frameworks offering basic, advanced, and BLAST sequence search options. Furthermore, precise annotations of peptides enable users to explore CRPs based on their specific activities (anticancer, no effect, insignificant effect, carcinogen, and others) and their effectiveness (rate and IC_50_) under cancer conditions, specifically within individual organs. This unique property facilitates the construction of robust training and testing datasets. Additionally, PeptiHub offers 1141 features with the convenience of selecting the most pertinent features to address their specific research questions. Features include aaindex1 (in six main subcategories: alpha propensities, beta propensity, composition indices, hydrophobicity, physicochemical properties, and other properties), amino acid composition (Amino acid Composition and Dipeptide Composition), and Grouped Amino Acid Composition (Grouped amino acid composition, Grouped dipeptide composition, and Conjoint triad) categories. These utilities not only speed up machine learning-based peptide design but also facilitate peptide classification.

**Database URL**: https://bioinformaticscollege.ir/peptihub/

## Introduction

**Figure 1.**
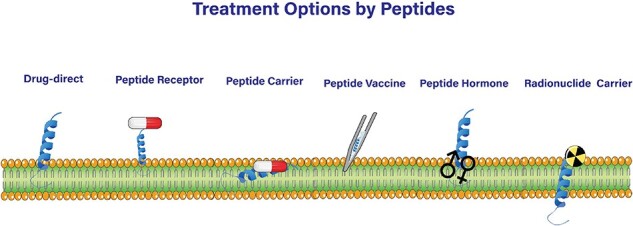
Peptide-Based Strategies for Cancer Treatment.

Anticancer peptides (ACPs) have emerged as a promising class for treating different types of cancers, including solid tumors and hematological malignancies [1, 2]. A major advantage of ACPs over traditional chemotherapy is their ability to selectively target and eliminate cancer cells, thereby avoiding significant adverse effects in vital organs such as the kidneys, gastrointestinal system, lungs, heart, and nervous system [3] associated with chemotherapy [4, 5]. This advantage is due to the unique mechanisms of action exhibited by ACPs, such as their ability to disrupt the cell membrane, induce apoptosis, and modulate the immune system, which impedes the development of drug-resistant cancer cells [6]. ACPs can also be applied in immunotherapies by enhancing the immune response against cancer cells, activating and increasing the number of immune cells including T cells and NK cells, and regulating immune checkpoints [7, 8]. Additionally, ACPs can be applied as peptide vaccines that hold epitopes of highly immunogenic antigens such as the Her2 receptor [9]. ACPs may not have therapeutic benefits on their own, but they can serve as drug conjugates [10, 11] to enhance the targeted delivery of anticancer agents into cells with improved tumor specificity [12, 13] (Fig. 1).

ACPs exert their effects through other mechanisms [14] as well. For example, they cause cancer cells to undergo both types of intrinsic and extrinsic apoptosis by targeting apoptotic components in cancer cells with minimal damage to the surrounding tissue [15–17]. Another mechanism of ACP action is the suppression of angiogenesis, which is vital for tumor growth [18], by inhibiting the angiogenesis-related pathways [19] (Fig. 1).

All of the aforementioned anticancer mechanisms fall into the cell-penetrating peptides category [20–23]. ACPs with no cell-penetrating potential are used in peptide receptor radionuclide therapy. In this strategy which provides a site-directed targeted therapy, a radiation dose is delivered to tumors by radiolabeled peptides [24] (Fig. 1).

ACPs can be classified into four types based on their conformation: α-helical ACPs, the most common form, are simple in structure but have drawbacks such as toxicity to normal cells. In contrast, β-pleated sheet ACPs are more complex and have lower toxicity to normal tissue cells but their anti-tumor activity is generally lower than that of α-helical ACPs. Random coil ACPs lack a typical secondary structure and are usually rich in proline and glycine. They have a lower killing effect on normal cells than other types of ACPs, but their inhibitory effect on tumor cells is worse than that of α-helical and β-pleated sheet ACPs. Cyclic ACPs are closed peptides that are more stable than linear structures, have a strong inhibitory effect on cancer cells with lower toxicity than other ACPs, and may be a useful reference for modifying ACPs effectively [25, 26]. Therefore, there is still a need to design new peptides that can overcome these limitations and provide better therapeutic outcomes.

Despite the development of vaccines and drugs such as Cervarix, Gardasil [27], Lutathera [28], Padcev [29], and Polivy [30], anticancer peptides have limitations in terms of their efficacy, specificity, and toxicity. Therefore, there is a need to design new peptides that can overcome these limitations and provide better therapeutic outcomes. In addition, the development of new anticancer peptides can lead to the discovery of new mechanisms of action and new targets for cancer therapy, which can ultimately lead to the development of more effective and targeted treatments for cancer. Accordingly, establishing a database of anticancer peptides can be beneficial for several reasons. First, it can provide a comprehensive resource for researchers and clinicians to access information about the properties and mechanisms of action of different anticancer peptides. This can aid in the development of new peptides with improved efficacy, specificity, and safety profiles. Secondly, a database can facilitate the identification of potential targets for cancer therapy and aid in the discovery of new mechanisms of action for anticancer peptides. Thirdly, a database can help to standardize the nomenclature and classification of anticancer peptides, which can improve communication and collaboration among researchers and clinicians.

Several databases cover ACPs with various fields of data and computational tools, such as Peptipedia [31] and CancerPPD [32], while others focus on a specific kind of ACP, such as apoptosis-inducing anticancer peptides (ApInAPDB) [33] and tumor neoantigen peptides (dbPepNeo) [34]. However, they suffer from some loss such as non-experimentally approved data, limited coverage, incomplete data, lack of integration with other resources such as protein databases, limited 3D structure, lack of experimentally approved negative dataset, and limited/lack of feature generating tools. Notably, the scarcity of experimentally validated non-ACPs challenges the creation of negative datasets for machine-learning-based approaches.

In this study, we provide PeptiHub, an experimentally approved manually curated publicly available database with precise and reliable literature extracted data and unique integration utilities and peptide features that facilitate the machine learning-based peptide design and classification. The maintenance of PeptiHub will prove to be advantageous to the cancer research community.

## Methodology

### Data collection, curation, and integration

The process of collection of data was initiated by exploring literature by different search engines such as Scopus, Google Scholar, Science Direct, PubMed, and Google using keywords: ‘anticancer peptides’, ‘peptide’ AND ‘cancer’, ‘peptide drug’, ‘peptide’ AND ‘tumor’. We have also conducted separate searches for the term ‘peptide’ in combination with each type of cancer to gather a comprehensive understanding of the potential application of peptides in cancer research. We also collected the CRP sequences from previously reported databases such as Peptipedia [31] and CancerPPD [32] containing data mining-extracted sequences and solely ACPs (positive dataset), respectively. Moreover, we also monitored the sequences in well-known available datasets ACP240 and ACP740 which were introduced by Yi *et al*. [35]. These datasets originated from Chen *et al*.’s work [36] in which ACPs (positive dataset) were obtained from the experimentally validated anticancer sequences that Hajisharifi *et al*. [37] collected from literature and the antimicrobial peptide database (https://aps.unmc.edu/). The negative dataset (non-ACPs), however, had no wet proof and it had been constructed based on the idea that most ACPs are secretary [38]. Therefore, Chen *et al*. assumed that non-secretary peptides deposited on UniProt server (UniProt) were non-ACPs. Therefore, it can be concluded that PeptiHub provides experimentally approved non-ACPs for the first time.

ACP sequences that were experimentally validated, along with relevant experimental information such as condition, experiment, cell line, experimental organism, organ, mechanism of action, target receptor, stage of disease, dose, IC_50_, rate, conjugate/carrier, organism of origin, and reference were meticulously extracted and compiled.

In addition, complementary data such as common name, original protein, match positions, UniProt ID, PDB structure (available on Uniprot revealed by either nuclear magnetic resonance spectroscopy (NMR)/X-ray or prediction), and RCSB ID were checked, manually obtained, and added to the data.

### Database technology stack

In the implementation of PeptiHub, we employed a diverse set of programming languages and methodologies to develop a sophisticated and user-friendly database. The backend infrastructure was constructed with the aid of PHP, a versatile server-side scripting language renowned for its efficiency and flexibility. On the front end, PeptiHub was designed using HTML, CSS, and JavaScript, laying the groundwork for an intuitive user interface. To optimize responsiveness, we seamlessly integrated Bootstrap, a widely recognized front-end framework. The accurate visualization of peptides was achieved using NGL viewer [39]. Furthermore, the integration of Ajax technology enabled real-time data retrieval, enhancing the platform’s interactivity and efficiency.

We utilized MySQL as the foundation of our data storage infrastructure. Furthermore, we implemented a tailor-made content management system (CMS) created with PHP. This CMS acts as an intuitive interface for curators to seamlessly input and oversee peptide data in the database. By leveraging this CMS, we optimize data entry, validation, and upkeep procedures, guaranteeing precise and current information at all times.

## Homology modeling

Peptides with no available 3D experimental or predicted structures on UniProt were subjected to homology modeling using SWISS-MODEL [40] and PEP-FOLD3 [41].

## Results and discussion

### Database content

**Figure 2.**
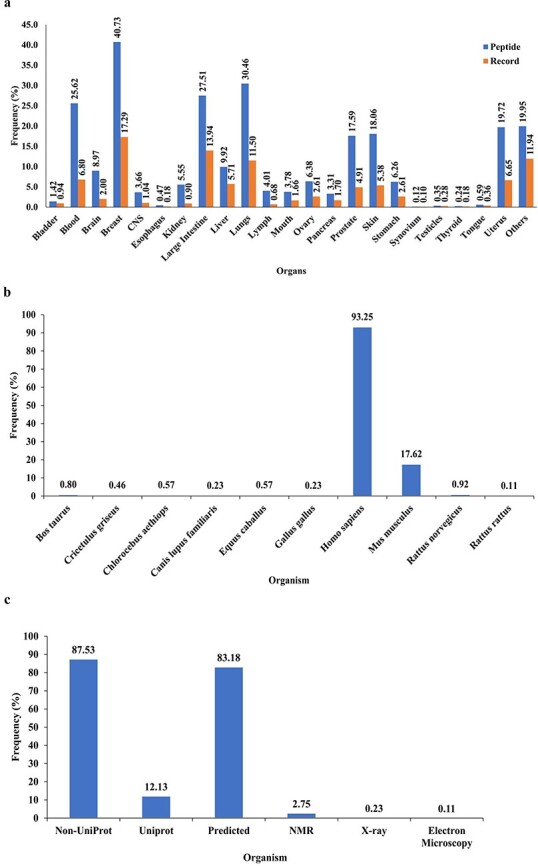
The overall content of PeptiHub.

PeptiHub is a comprehensive database that comprises 7237 records of 874 unique peptides, with a length ranging from 2 to 172 residues. The content of PeptiHub reveals that unique sequences of CRPs have been predominantly studied against tumors in the breast, blood, large intestine, and lungs on 438 different cell lines, thereby providing a preliminary insight into the gaps where researchers can design new therapeutic peptides (Fig. 2a). Notably, 93.25% of the unique sequences were tested on humans, followed by *Mus musculus*, with 17.62% of CRPs (Fig. 2b).

In terms of integration with other databases, Fig. 2c indicates that only 12.13% of peptides had a record on UniProt, with 3.09% having available 3D structure generated by NMR, X-ray, and electron microscopy. The remaining 83.18% of the sequences were subjected to homology modeling.

Furthermore, it is noteworthy that 755 peptides have demonstrated anticancer effects in diverse conditions, while 120 peptides have been identified as non-effective in the literature, as they exhibit no difference compared to the control. This is comparable to 181 sequences that have an effect on tumors, but it is not statistically significant. Additionally, 14 peptides have shown a converse effect and have caused cancer in healthy cell lines, while 38 peptides have no drug role in cancer treatment. Given the variability of cell lines and assay conditions, it is highly recommended that users carefully monitor the cell line, dose, and organism of the test before incorporating sequences into their training datasets.

### PeptiHub utility

**Figure 3.**
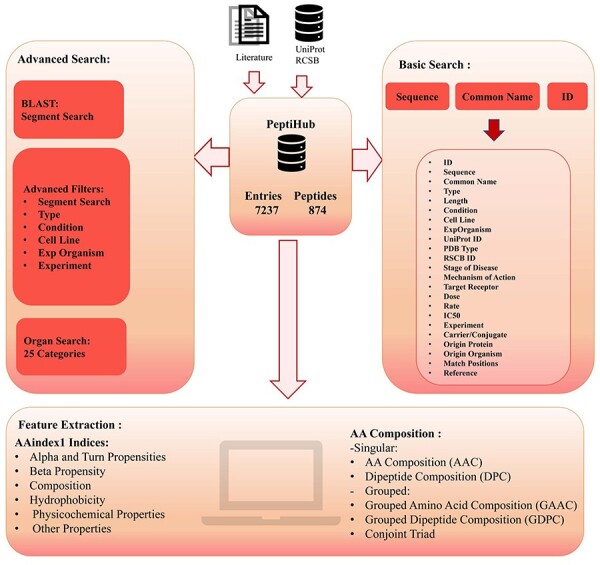
The overall components and utilities of PeptiHub.

The overall organization of Peptihub is shown in Fig. 3. The standout features of Peptihib can be categorized into two main groups:

#### Precision

Accurate information on various factors such as IC_50_ values, cell types, experimental techniques and outcomes, peptide origins, mechanism of action, dosages, protein characteristics, cancer classifications, and peptide sources for ACPs is crucial for gaining a comprehensive insight into the effectiveness of a peptide candidate within biological systems. Existing databases often lack this level of detail and precision. A substantial portion of peptides available on other platforms are suggested through data mining techniques. Upon thorough examination of these peptides, we discovered a lack of experimental verification regarding the anticancer properties of many sequences. In contrast, PeptiHub’s data are firmly grounded in experimental evidence, and the detailed dosages used in an experimental trial, along with their corresponding results, are meticulously documented in separate records. This provides researchers with the necessary information to pinpoint the optimal treatment concentration for their experiments but also offers insights into the safety profile of a specific peptide. It is crucial to note that the PeptiHub developers abstain from drawing definitive conclusions regarding the anticancer properties of a peptide. This caution is warranted due to the complex nature of experimental outcomes, where a peptide demonstrating anticancer efficacy at one concentration could potentially exhibit carcinogenic properties at another. Hence, rather than making definitive claims about anticancer effects, PeptiHub offers detailed information on the diverse effects observed at different concentrations. This unique approach sets PeptiHub apart from other sources, offering researchers the flexibility to define the anticancer properties according to their specific research preferences. For example, one research study may choose to categorize both ‘no effect’ and ‘insignificant’ types as a negative dataset, while another study may opt to include only the ‘no effect’ type.

#### Machine learning facilities

Peptihub offers 1141 calculated features for each peptide. This allows for easy access to essential features necessary for machine learning-based tasks. By utilizing these comprehensive datasets, researchers can greatly enhance their work in advancing the field of cancer therapy and drug design. Significantly, the lack of experimentally validated non-ACPs in current databases poses a challenge for constructing negative datasets in machine learning applications. This challenge is effectively addressed by PeptiHub.

### Basic search

**Figure 4.**
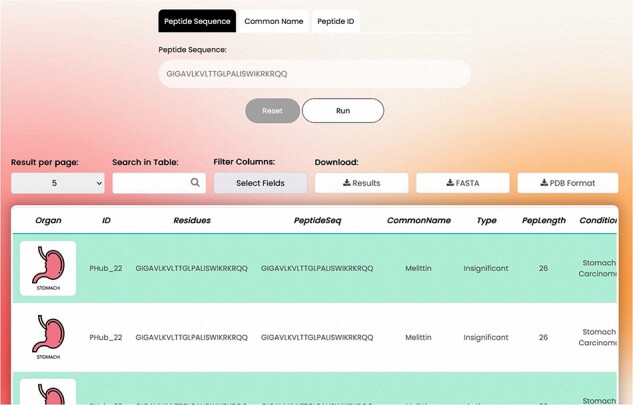
Basic search.

The overall appearance of the basic search page is shown in Fig. 4.

**Search Tabs**: The detailed information of a peptide can be explored using the PeptiHub homepage which contains basic search tabs based on a peptide’s sequence (in one-letter format), common name, and PeptiHub ID as a query. Using the downside scroll bar, a table of the following columns appears:

- Organ: The organ that the experiment was conducted on, shown in a schematic picture for easier follow-up.
- Type: The experimentally determined activity of a peptide falls into the following categories:
  1. Anticancer: peptides whose statistically significant anticancer effect is shown by an assay.
  2. No effect: peptides that showed no antitumor activity in the assays compared to the control group.
  3. Insignificant: peptides that had statistically insignificant anticancer activity compared to the control group. It is up to the esteemed user whether to consider insignificant peptides as non-anticancer or weak anticancer.
  4. Carcinogen: peptides used in cancer assays since they were expected to have anticancer activity but they showed converse results and enhanced cancer condition.
  5. Others: peptides that were involved in cancer research but had no direct anticancer effect. They were usually applied as carriers for other drugs. In this case, the co-treatment/ conjugate compound is also provided in a separate field.
- PepLength: The number of residues in the peptide sequence.

- Condition: The experimental condition under which the peptide was tested (illustrated schematically). These terms are implemented based on the International Classification of Diseases for Oncology, 3rd edition (ICD-O-3), dated 29 April 2022. This standardized classification system allows for the inclusion of specific classes of cancers that may implicate a single organ, thereby providing users with standard information on various cancer types associated with a particular organ.
  - Cell Line: The cell line used in the experiment. The cell lines are mentioned based on the Cellosaurus, a reputable Cell line encyclopedia (https://www.cellosaurus.org/), provided by the Swiss Institute of Bioinformatics.
  - ExpOrganism: The organism used in the experiment provided in scientific name.
  - PeptideUniProtID: The UniProt ID of the peptide, showing that the exact sequence of the peptide has an ID in the UniProt database.
  - Peptide PDB Type: A PDB file is a file format that contains information about the 3D structure of a biological molecule like proteins, nucleic acids, and complex assemblies. These files typically include the following information:

– Atomic coordinates of the atoms in the molecule.
– Information about the type and connectivity of atoms.
– Structural information such as bond lengths and bond angles.
– Metadata describing the experimental method used to determine the structure.
– Information about any ligands or cofactors bound to the molecule.

Researchers use PDB files to study the structure and function of biological molecules, to design drugs, and to understand molecular interactions.

All peptides in PeptiHub are provided with a PDB format but extracted from different methods including ‘NMR’, ‘X-ray’, and ‘electron microscopy’. Nonexperimental models are also determined as ‘predicted’.

- Peptide RCSB ID: PDB ID is a special code for identifying 3D structures of biological molecules on the RCSB Protein Data Bank (https://www.rcsb.org/).
- Stage of disease: The stage of cancer at which the experiment was conducted.
- Mechanism of action: the mechanism by which the peptide exerts its effect. For non-ACPs, this column identifies that the peptide has no anticancer effect through the mentioned mechanism of action.
- Target receptor: the experimentally identified receptor of the peptide.
- Dose: The dose of the peptide used in the experiment with its unit in the next column (Unit1). Researchers may find the effective/ineffective dose of their peptide of interest here.
- Rate: The rate of experimentally shown anticancer activity.
- IC_50_: The half maximal inhibitory concentration of the peptide accompanied by the corresponding unit in the next column (Unit 2).
- Experiment: The ‘Experiment’ column aims to identify the assay used to demonstrate the peptide’s activity. However, this information can also be intricate regarding the administration period. For example, a peptide might exhibit no effect after 24 h but demonstrate anticancer activity after 48 h. To offer maximum precision, PeptiHub includes the administration period separated by a space, allowing users to filter peptides based on their specific research requirements.
- Carrier/conjugate: The conjugate/carrier which is co-treated by the peptide.
- Origin protein: Name of the protein/peptide that an arbitrary sequence is derived from.
- Origin UniProt: UniProt ID of the protein/peptide that an arbitrary sequence is derived from.
- Origin organism: The organism from which the peptide was derived.
- Match positions: the positions in the original protein that match the peptide sequence. The origin categories are provided according to the UniProt database.
- Reference: the published original article for the result.

### 3D structure and visualization

PeptiHub provides 3D structures of all entries in PDB format. Peptides with experimentally revealed conformation had RCSB PDB ID (RCSB PDB: Homepage). Since there were abundant peptides with no available PDB structures, we provided their computational models by homology modeling and indicated them by ‘Predicted’. Fastq format is also available for each entry. Moreover, the resulting records can be downloaded in CSV format for more convenience.

### Advanced search

**Figure 5.**
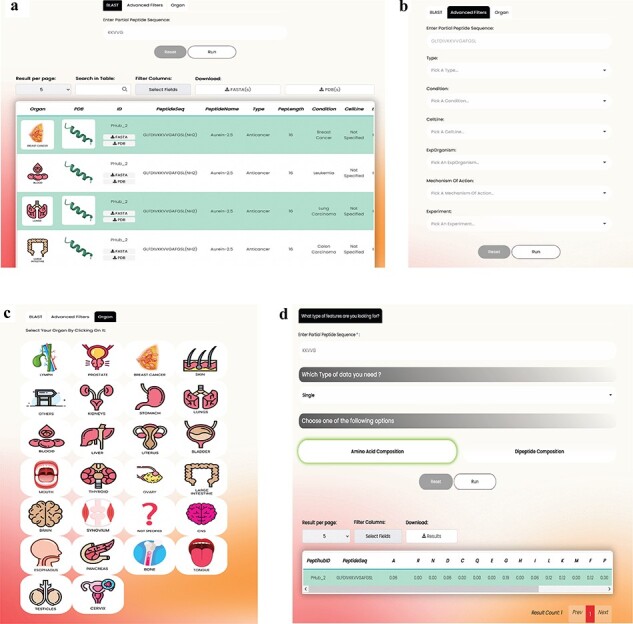
The overall view and the utilities offered in (a) BLAST, (b) Advanced filters, (c) Organ tabs on the Advanced search page as well as (d) features available on the feature extraction page.

The advanced search section offers users a range of additional utilities compared to the basic search, providing greater control over their exploration compared to the basic search (Fig. 5a, b, and c). These include:

- BLAST: By using the BLAST tab, users can search for an arbitrary sequence, resulting in a list of peptides that contain the query. In contrast, the basic search page only explores exact matches of entire sequences within the database.
- Advanced Filters: Clicking on the Advanced Filters button reveals a selection of additional filters that users can apply to refine their search. These filters encompass various options such as specific peptide Types, Conditions, Cell Lines, Experiments, Experiment Organism, and Mechanism of Action.
- Organ: There might be several cancer terms for a single organ. For instance, ‘breast carcinoma’ and ‘breast adenocarcinoma’ represent two distinct types of breast cancers. Recognizing the importance of ease of access to information, we have also introduced the ‘Organ’ tab on the advanced search page. This feature enables users to explore and access all ACPs about different cancer subcategories within a specific organ. By implementing these enhancements, we aim to streamline the search process, improve data reliability, and enhance the overall user experience on PeptiHub.

In addition, users can select the output columns. Furthermore, PDB and FASTQ files of all output records are readily accessible, permitting both simultaneous and individual retrieval.

### Feature extraction

PeptiHub offers an extensive number of numerical descriptions, known as features [42], for all peptides. These features could significantly contribute to research inquiries, especially in predicting peptide functions and advancing peptide drug design. Within PeptiHub, users are provided with the convenience of selecting the most pertinent features to address their specific research questions (Fig. 5d). These readily accessible features can be effectively employed in training machine learning models for peptide design and constructing peptide libraries. The distinguished collection of features in PeptiHub derives from aaindex1, sourced from the AAindex server [43] (https://www.genome.jp/aaindex/). All indices are categorized into six distinct classes, encompassing alpha and turn propensities, beta propensity, composition, hydrophobicity, physicochemical properties, as well as other properties done by the AAindex developers [43] based on Tomii’s study [44].

In addition, amino acid composition is also provided in two distinct schemes:

**Amino acid compositions**:

– Amino acid composition (AAC): This feature quantitively represents the frequency of each amino acid in peptide sequences, consisting of 20 distinct descriptors.
– Dipeptide composition (DPC) [45]: DPC captures the residue-pair frequency of amino acids within a sequence, comprising 400 descriptors.

**Figure 6.**
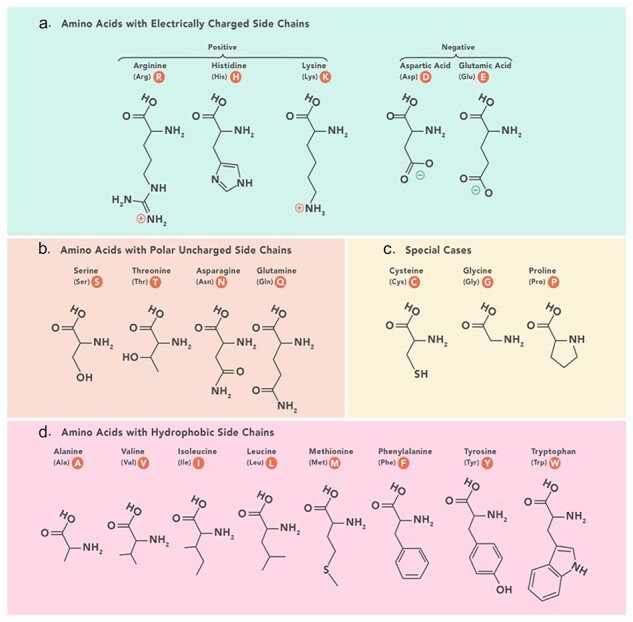
Amino acids physicochemical classes based on which grouped features are categorized in PeptiHub.

**Grouped amino acid composition**:

The grouped amino acid composition method represents the composition of amino acids in a peptide sequence based on predefined physicochemical categories including positively charged, negatively charged, polar uncharged, hydrophobic, and special amino acids (Fig. 6). Instead of considering the composition of individual amino acids, grouped amino acid composition considers amino acids with similar physicochemical properties.

- Grouped amino acid composition (GAAC): GAAC provides information about the overall distribution of the amino acid groups in the peptide sequence. This feature comprises five distinct descriptors.

- Grouped dipeptide composition (GDPC): GDPC extends GAAC by considering pairs of adjacent amino acid residues in the peptide sequence. It groups the 400 DPC dipeptides into smaller sets. This feature comprises 25 distinct descriptors.

- Conjoint triad [46]: Conjoint triad considers the composition and sequential order of three adjacent amino acids (tripeptides) based on the predefined physicochemical categories of amino acids. This feature comprises 125 distinct descriptors.

PeptiHub is committed to the continuous enhancement of the data by actively screening and incorporating novel ACPs supported by empirical evidence to stay as a dynamic resource for the cancer research community. As we look ahead, the introduction of new tools such as prediction and mutation tools, alongside ML-based models, will empower researchers to evaluate and enhance the anticancer potential of diverse peptide sequences. Furthermore, our vision for enhanced integration with prominent databases like DAVID promises to create a more interconnected and comprehensive research environment, fostering collaboration and accelerating advancements in the field of peptide-based anticancer therapies.

## Data Availability

PeptiHub can be freely accessed at the URL: https://bioinformaticscollege.ir/peptihub/.
